# Curcumin Protects Osteoblasts From Oxidative Stress-Induced Dysfunction via GSK3β-Nrf2 Signaling Pathway

**DOI:** 10.3389/fbioe.2020.00625

**Published:** 2020-06-16

**Authors:** Xumin Li, Yang Chen, Yixin Mao, Panpan Dai, Xiaoyu Sun, Xiaorong Zhang, Haoran Cheng, Yingting Wang, Isaac Banda, Gang Wu, Jianfeng Ma, Shengbin Huang, Tim Forouzanfar

**Affiliations:** ^1^Department of Prosthodontics, School and Hospital of Stomatology, Wenzhou Medical University, Wenzhou, China; ^2^Department of Oral Implantology and Prosthetic Dentistry, Academic Centre for Dentistry Amsterdam, MOVE Research Institute, University of Amsterdam and Vrije University Amsterdam, Amsterdam, Netherlands; ^3^Institute of Stomatology, School and Hospital of Stomatology, Wenzhou Medical University, Wenzhou, China; ^4^Department of Oral and Maxillofacial Surgary/Pathology, Amsterdam UMC and Academic Centre for Dentistry Amsterdam, Amsterdam Movement Science, Vrije Universitetit Amsterdam, Amsterdam, Netherlands; ^5^Laboratory for Myology, Amsterdam Movement Sciences, Faculty of Behavioral and Movement Sciences, Vrije Universiteit Amsterdam, Amsterdam, Netherlands; ^6^Department of Stomatology, Taizhou Hospital, Wenzhou Medical University, Linhai, China; ^7^Department of Periodontology, School and Hospital of Stomatology, Wenzhou Medical University, Wenzhou, China; ^8^Department of Endodontics, School and Hospital of Stomatology, Wenzhou Medical University, Wenzhou, China

**Keywords:** curcumin, oxidative stress, osteoblast, dysfunction, GSK3β, Nrf2

## Abstract

Osteoblasts dysfunction, induced by oxidative stress (OS), is one of major pathological mechanisms for osteoporosis. Curcumin (Cur), a bioactive antioxidant compound, isolated from Curcumin longa L, was regarded as a strong reactive oxygen species (ROS) scavenger. However, it remains unveiled whether Cur can prevent osteoblasts from OS-induced dysfunction. To approach this question, we adopted a well-established OS model to investigate the preventive effect of Cur on osteoblasts dysfunction by measuring intracellular ROS production, cell viability, apoptosis rate and osteoblastogenesis markers. We showed that the pretreatment of Cur could significantly antagonize OS so as to suppress endogenous ROS production, maintain osteoblasts viability and promote osteoblastogenesis. Inhibiting Glycogen synthase kinase (GSK3β) and activating nuclear factor erythroid 2 related factor 2 (Nrf2) could significantly antagonize the destructive effects of OS, which indicated the critical role of GSK3β-Nrf2 signaling. Furthermore, Cur also abolished the suppressive effects of OS on GSK3β-Nrf2 signaling pathway. Our findings demonstrated that Cur could protect osteoblasts against OS-induced dysfunction via GSK3β-Nrf2 signaling and provide a promising way for osteoporosis treatment.

## Introduction

Osteoporosis, a systemic degenerative disease during aging, is associated with increased fragility and fracture risk of bone. It is characterized by decrease of bone mass and deterioration of bone architectural structure due to the imbalance between bone formation and resorption (Fonseca et al., 2014). Roughly nine million osteoporotic or fragility (low-trauma) fractures occur worldwide per year (Johnell and Kanis, 2006). In developed countries, around one in three women and one in five men aged 50 years or older will have a fragility fracture during their remaining lifetime, thereby significantly affecting their life quality and expectancy (Shepstone et al., 2018). However, hitherto, the mechanisms for osteoporosis are not completely unveiled and there is still a lack of efficacious treatment strategies helping bone tissue regeneration under this circumstance.

A great line of evidences reveal that oxidative stress (OS) is a crucial pathogenic factor for osteoporosis (Manolagas, 2010; Molnar et al., 2016). Our previous meta-analysis study demonstrates that the decrease of bone mineral density (BMD) in osteoporotic postmenopausal women is closely related to the status of OS (Zhou et al., 2016). In animal models, significantly decreased endogenous antioxidant defense and increased oxidative damages are detected in osteoporotic bone tissue induced by ovariectomy (Mohd Effendy et al., 2015; Serizawa et al., 2016). Furthermore, approaches to attenuate OS have been found to effectively delay or inhibit the progress of osteoporosis (Li et al., 2017; Zhu et al., 2018). All these findings indicate the paramount importance of OS in the pathogenic process of osteoporosis.

Osteoblasts play a crucial role in bone maintenance and regeneration. Its cellular interactions with osteoclasts are essential to regulate bone mass homeostasis as well as bone quality (Nevius et al., 2015; Maria et al., 2018). During osteoblastogenesis, mesenchymal stem cells are first osteogenically committed and become preosteoblasts. Thereafter preosteoblasts undergo the process of osteoblastogenesis during which the alkaline phosphatase (ALP) activity (a marker for an early osteoblastogenic differentiation) and osteocalcin (OCN) expression (a marker for a late osteoblastogenic differentiation) in cells will be significantly enhanced, finally leading to cell matrix mineralization. During this process, the expression of osteoblastogenic genes, such as Runt-related transcription factor 2 (Runx2), Collagen Iα (Col I), will also be significantly enhanced. It has been established that the reduction of osteoblasts viability and function during aging leads to the imbalance of bone formation and bone resorption, which is significantly associated with the onset and progression of osteoporosis (Farr et al., 2017; Xu et al., 2017). Persistent or prolonged OS can not only inhibit proliferation (Li et al., 2009) and differentiation (Zhong et al., 2009) of osteoblasts but also induce their apoptosis (Almeida et al., 2010; Wu et al., 2011; Mao et al., 2018), which results in bone loss during osteoporosis. Therefore, bioactive agents to attenuate OS damages to osteoblasts may be promising in treating osteoporosis (Hendrickx et al., 2015; Mao et al., 2018).

One of such bioactive agents is Curcumin (Cur), the major active ingredient of turmeric plant (Curcumin longa L). Cur has been long recognized as an anti-inflammatory and anti-bacterial agent (Yodkeeree et al., 2009; Feng et al., 2019) to treat various chronic inflammatory diseases (Goel et al., 2008). Moreover, emerging evidences reveal that Cur can protect liver and kidney from drugs- or toxins-induced acute or chronic injury by scavenging reactive oxygen species (ROS) and improving anti-oxidative ability (Sahin et al., 2012; Tokac et al., 2013; Wu et al., 2017). Further report demonstrates that Cur improves bone microarchitecture and enhances mineral density in amyloid precursor protein (APP)/presenilin (PS1) transgenic mice (Yang et al., 2011), a transgenic mouse representing the pathological changes of senile dementia and osteoporosis. On the one hand, such an effect of Cur is attributed to its capacity of suppressing osteoclastogenesis by inhibiting receptor activator of nuclear factor-κB ligand (RANKL) signaling, an essential signaling for the differentiation of bone-resorbing osteoclasts (Folwarczna et al., 2010; Kim et al., 2011; Hussan et al., 2012; Cho et al., 2013). On the other hand, in our previous study, we also showed that Cur attenuated OS-induced apoptosis of human osteoblastic cell (Saos-2) by preserving the mitochondrial functions and upregulating phosphorylated protein kinase B (Akt)-Glycogen synthase kinase 3β (GSK3β) signaling (Chao et al., 2017). However, hitherto, it remains unknown whether Cur can antagonize oxidative damages so as to maintain the osteogenic function of osteoblasts under the OS attack.

Glycogen synthase kinase 3β is a serine/threonine kinase that is involved in cell apoptosis, inflammatory reactions as well as OS related diseases (Qi et al., 2015). GSK3β can significantly down-regulate the expression of nuclear factor (erythroid-derived 2)-like 2 (Nrf2), a key transcription factor accounting for cell antioxidant defense (Zhuang et al., 2019). Nrf2 has been suggested to be a novel target to slow down the progression of bone degenerative disorders (Jiang et al., 2018; Olagnier et al., 2018). However, whether GSK3β-Nrf2 signaling pathway is involved in regulating OS-induced osteoblasts dysfunction remains unknown.

In this study, we adopted a well-established cell oxidative injury model aiming (1) to investigate the effects of Cur on OS-induced osteoblasts dysfunction and (2) to verify whether GSK3β-Nrf2 signaling pathway mediated the protective effect of Cur.

## Materials and Methods

### Experiment Design

We treated a mouse preosteoblast cell line (MC3T3-E1 cells) with hydrogen peroxide (H_2_O_2_) to establish an oxidative injury model. Cur or N-acetyl-L-cysteine (NAC) was preincubated as antioxidant. The intracellular ROS production, cell viability, apoptosis rate and osteoblastogenesis markers were measured and compared among different groups. After detecting the level of phosphorylated-GSK3β and Nrf2, the specific chemical GSK3β inhibitor 2-Methyl-4-(phenylmethyl)-1,2,4-thiadiazolidine-3,5-dione (TDZD-8) and Nrf2 activator tertiary butylhydroquinone (tBHQ) were added to the model to further confirm the role of this signaling pathway in OS suppressing, cell viability protecting and osteogenesis promoting.

### Cell Culture

MC3T3-E1 cells (obtained from American Type Culture Collection, ATCC) were cultured in α-minimum essential medium (α-MEM) supplymented with 10% fetal bovine serum (FBS) and 100 U/mL penicillin, and 100 U/mL streptomycin. This basic medium was replaced every 3 days. For osteogenetic differentiation induction, when, β-glycerophosphate (5 mM, Sigma, United States) and ascorbic acid (100 mg/mL, Sigma, United States) were added to basic culture medium after cells at 80% confluence, and differentiation medium was changed every 3 days.

### Cell Treatment

The different treating condition of the compounds were as follows: H_2_O_2_ (0.1–1 mM, Sigma, United States) for 6 h, Cur (0.25 μM, Sigma, United States) for 24 h, TDZD-8 (5 μM, Sigma, United States) for 1 h, tBHQ (5 μM, Sigma, United States) for 1 h, NAC, the widely used antioxidant for positive control (2.5 mM, Sigma, United States) for 1 h, according to previous studies (Dai et al., 2017; Cuadrado et al., 2018; Huang et al., 2019) and our preliminary data (data not shown). Cells were treated with or without H_2_O_2_ and the indicated compounds for various conditions in basic medium or osteogenesis differentiation medium. The final concentration of dimethyl sulfoxide (DMSO, Sigma, United States) was diluted to less than 0.5% in all experiments.

### Cell Viability Assay

Apoptosis of MC3T3-E1 cells were detected by 3(4,5-dimethylthiazol-2-yl)-2,5-diphenyltetrazolium (MTT, Sigma, United States) bromide method. MC3T3-E1 cells (1 × 10^4^ cells/well) were plated on 96-well plates and treated as indicated above. Cell viability at 24, 48, and 96 h was measured as previously described (Dai et al., 2017).

### Measurement of Apoptosis by Flow Cytometry and Deoxynucleotidyl Transferase dUTP Nick End Labeling Assays

Apoptosis of MC3T3-E1 cells were detected by Annexin V-fluorescein isothiocyanate (FITC, 5 μl, Thermo Fisher Scientific, United States); propidium iodide (PI; 10 μl, Thermo Fisher Scientific, United States) was used to determine cell necrosis. After treatment, cells were harvested and resuspended influorochromes at 37°C, then incubated in the dark for 15 min. Cytofluorometric analysis was using a FACScan (Becton Dickinson, NY, USA).

Apoptosis was also evaluated by the terminal transferase dUTP nick end labeling (TUNEL) staining (Roche, Switzerland), following the manufacturer’s instructions. Cells were incubated on different coverslips. After treatment, the cells were fixed in 4% paraformaldehyde (PFA), gently washed with PBS and mixed with 0.2% Triton X-100. Samples were incubated with newly prepared TUNEL assay solution 1h, then counterstained with 4′, 6-diamidino-2-phenylindole (DAPI, Sigma, United States) 5 min in the dark. Cells were observed by fluorescence microscope (Leica TCS SPE, Germany), and the nucleus fluoresced brightly green were considered as TUNEL positive cell. Percentages of TUNEL positive cells were calculated by counting 300 cells in random fields.

### ROS Assay

2′,7′-Dichlorofluorescin Diacetate (DCFH-DA, Thermo Fisher Scientific, United States) were used to assess ROS generation. MC3T3-E1 cells (1 × 10^4^ cells/well) were seeded in chamber slides. Cells were treated with or without H_2_O_2_ (0.75 mM, 6 h), pre-incubating with Cur for 24 h or the positive control NAC for 1 h. After treatments, MC3T3-E1 cells were incubated with 10 μM DCFH-DA for 30 min at 37°C and fixed in 4% PFA for 30 min at room temperature. After being washed with PBS, the fixed cells were stained with 20 μg/mL DAPI in the dark for 5 min at room temperature. Then the fluorescence of cells was detected by a fluorescence microscope and quantified by NIH Image J software (public domain).

### ALP Activity Assay and ALP Staining

MC3T3-E1 cells (3 × 10^4^ cells/well) were seeded on 48-well plates and stimulated with osteogenesis differentiation medium for 7 days. After treatment, ALP activity of the cell lysate was assayed by an ALP assay kit (Beyotime, China). In brief, 50 μL of sample was incubated for 10 min in a 96-well plate with 50 μL newly prepared work solution in 37°C. After stop of the reaction with 100 μL stop solution, the absorbance was measured at 405 nm with a micro-plate reader. Protein concentration was determined using BCA protein assay (Thermo Fisher Scientific, United States).

Alkaline phosphatase staining was performed by a standard protocol. Briefly, samples were fixed with 4% PFA for 30 min at 4°C and stained by a BCIP/NBT ALP color development kit (Beyotime, China) according to the manufacture’s instruction. After staining, cells were washed with deionized water three times.

### Mineralization Assay

MC3T3-E1 cells (3 × 10^4^ cells/well) were seeded on 48-well plates and were stimulated with osteogenic differentiation medium for 14 days. After osteogenic differentiation, the cells were gently washed twice with PBS and fixed with 4% PFA for 30 min at 4°C. Then, cells were stained with 0.1% Alizarin red (Sigma, United States) for 1 h at room temperature. Excess dye was then washed away with deionized water and each well was photographed and quantified by ImageJ.

### Quantitative Real-Time Polymerase Chain Reaction (rt-PCR)

Total RNA from MC3T3-E1 cells was extracted using Trizol reagent (Invitrogen, United States). cDNA synthesis was performed with 1 mg RNA using PrimeScript RT reagent Kit with gDNA Eraser (Takara, Japan) and quantified by measuring the absorbance at 260 and 280 nm. Samples were analyzed in triplicate. cDNA was amplified using following gene-specific primers listed in Table 1. PCR was then carried out for 30 cycles consisting 1min each for 94°C (denaturation), 60°C (annealing), and 72°C (elongation), and final extension was done at 72°C for 10 min using TB Green Premix Ex Taq (Takara, Japan) according to the manufacture’s instruction.

**TABLE 1:** Primers sequences for rt-PCR.

Gene	Forward primer (5′-3′)	Reverse primer (5′-3′)
Akp2 (ALP)	TGCCTACTTGTGTGGCGTGAA	TCACCCGAGTGGTAGTCACAATG
Osteocalcin (OCN)	AGCAGCTTGGCCCAGACCTA	TAGCGCCGGAGTCTGTTCACTAC
Collagen I (COL I)	ATGCCGCGACCTCAAGATG	TGAGGCACAGACGGCTGAGTA
Runt-related transcription factor 2 (Runx2)	CACTGGCGGTGCAACAAGA	TTTCATAACAGCGGAGGCATTTC
Glyceraldehyde-3-phosphate dehydrogenase (GAPDH)	TCAACAGCAACTCCCACTCTT	ACCCTGTTGCTGTAGCCGTATTCA

### Protein Extraction and Western Blot Analysis

After the indicated treatment, MC3T3-E1 cells were harvested and proteins from them were extracted using radioimmunoprecipitation assay buffer (RIPA) buffer (Sigma, United States). Equal amounts of protein were separated by SDS-PAGE and transferred into a polyvinylidene difluoride membrane. Concentration of primary antibodies are as follows: anti-phospho-GSK3β (1:4000, Cell Signaling, United States), anti-GSK3β (1:4000, Cell Signaling, United States), anti-Nrf2 (1:1000, Santa, United States), and anti-β-actin (1:8000, Sigma, United States). The following secondary antibody were horseradish peroxidase conjugated anti-mouse IgG antibody (1:4000, Invitrogen, United States) or anti-rabbit IgG antibody (1:4000, Invitrogen, United States), followed by the incubation of enhanced chemiluminescence (ECL) substrate. The immunoreactive bands intensities were quantified using ImageJ software and normalized with β-actin levels.

### Statistical Analysis

Data are described as mean ± SD (the standard deviation of the mean). All statistical analysis was performed with Statview software (SAS Institute, Version 5.0.1). Differences between groups were assessed by one-way analysis of variance (ANOVA) with Fisher’s *post hoc* test. Significant difference was accepted at *P* < 0.05.

## Results

### Cur Attenuated H_2_O_2_-Induced Apoptosis and ROS Generation in MC3T3-E1 Cells

**FIGURE 1:**
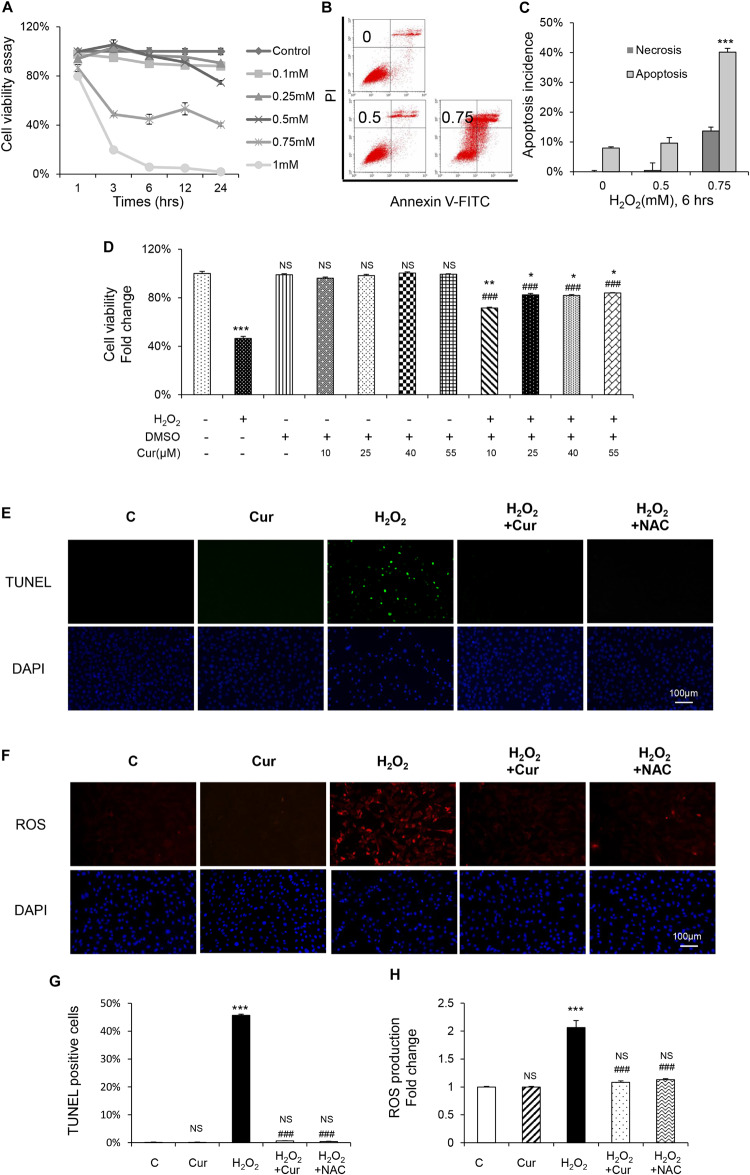
Cur attenuated H_2_O_2_-induced apoptosis and ROS generation in MC3T3-E1 cells. **(A)** MC3T3-E1 cells were treated with or without H_2_O_2_ in the basic medium. Cell viability was determined by MTT reduction in MC3T3-E1 cells in the presence of different concentration of H_2_O_2_ for 1, 3, 6, 12, 24 h. **(B,C)** The flow cytometric analysis of staining from control group, 0.5 and 0.75 mM H_2_O_2_ for 6 h. **(D)** Cur was added 24 h before H_2_O_2_. Cell viability was determined by MTT reduction in MC3T3-E1 cells in the presence of 0.10, 0.25, 0.40, and 0.55 μM Cur for 24 h with (+) or without (–) H_2_O_2_. **(E,G)** The cells were immunostained for TUNEL (green). DAPI staining was used to mark the position of the nuclei. Scale bars = 100 μm. **(F,H)** The cells were harvested and stained with DCFH-DA (red). Scale bars = 100 μm. Data are presented as the mean ± SD from at least three independent experiments. *p < 0.05, **p < 0.01, ***p < 0.001, versus C group; NS, non-significantly different from C group. ^#^*p* < 0.05, ^##^*p* < 0.01, ^###^*p* < 0.001, versus the H_2_O_2_ group; ns, non-significantly different from H_2_O_2_ group.

In order to investigate the antioxidant effect of Cur, MC3T3-E1 cells were exposed to H_2_O_2_ at different concentration with different enduring time according to our previous study (Dai et al., 2017). Compared with the vehicle group, H_2_O_2_ significantly decreased osteoblasts viability in the dose and time dependent way (Figure 1A). Flow cytometric analysis also showed a dose-dependent increasing of apoptosis. Early apoptosis was detected after the administration of 0.5 mM H_2_O_2_, and higher concentration of H_2_O_2_ induced late apoptosis with a slight increase of necrosis (Figures 1B,C). Treated with H_2_O_2_ at a concentration of 0.75 mM with 6 h was the half inhibitory concentration (IC) of MC3T3-E1 cells and can cause cell apoptosis to a certain degree. Therefore, this condition was chosen in the following experiments. Our results showed that Cur ranging from 0.01 to 1.0 μM was not cytotoxic to MC3T3-E1 cells. It reversed cell viability reduced by H_2_O_2_ and played its best role under the condition of 0.25 μM, pretreating 24 h (Figure 1D). Furthermore, the result of TUNEL staining (Figures 1E,G) indicated the decreased percentage of apoptotic cells by using Cur. In Figures F,H, we showed that the ROS level increased by H_2_O_2_ were attenuated by Cur. The effect of Cur was the similar to classical antioxidant NAC, which indicated that Cur had positive effect against OS.

### Cur Rescue H_2_O_2_-Induced Osteoblasts Dysfunction

**FIGURE 2:**
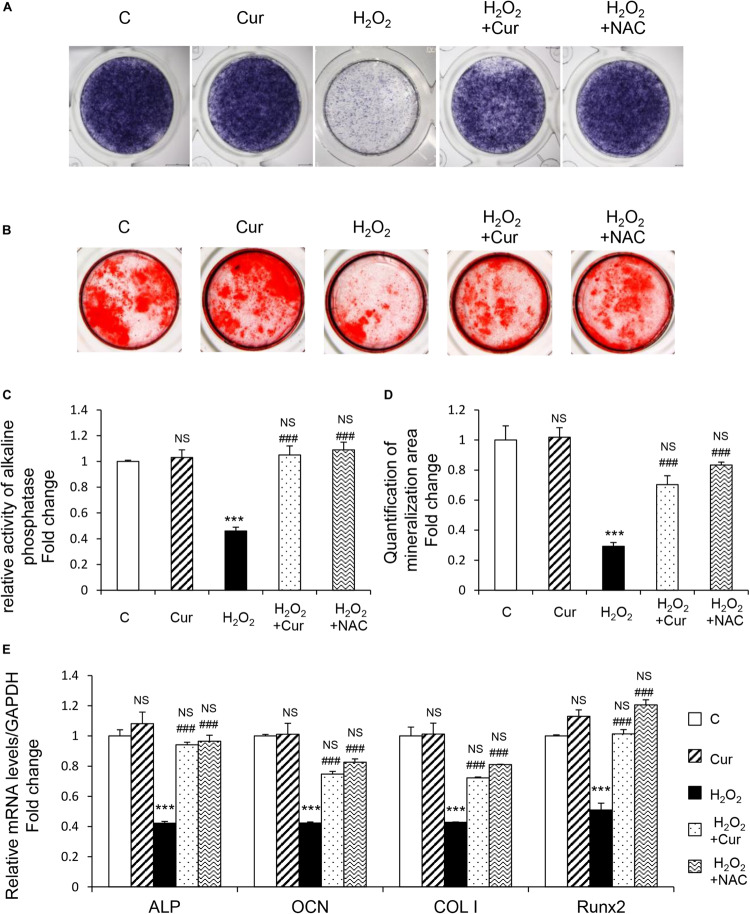
Cur rescue H_2_O_2_ induced osteoblast dysfunction. The MC3T3-E1 cells were treated in the osteogenesis differentiation medium with or without H_2_O_2_ and Cur. **(A)** After the cells cultured for a week and they were subjected to ALP staining in the indicated treatment groups. **(B)** The alizarin red staining of MC3T3-E1 cells showed the mineralizing matrix after cultured for 2 weeks. **(C)** ALP activity tested in MC3T3-E1 cells as indicated groups. **(D)** Mineralization area was evaluated by tests. **(E)** When cultured for 3 days the expression of osteogenic marker genes were analyzed. Data are presented as the mean ± SD from at least three independent experiments. *p < 0.05, **p < 0.01, ***p < 0.001, versus C group; NS, non-significantly different from C group. ^#^*p* < 0.05, ^##^*p* < 0.01, ^###^*p* < 0.001, versus the H_2_O_2_ group; ns, non-significantly different from H_2_O_2_ group.

MC3T3-E1 cells were supplemented with differentiation medium to initiate osteogenic induction. ALP, as the by-product of osteoblasts activity, was decreased by H_2_O_2_ and recovered by Cur during the differentiation process showed by the results of both ALP staining and activity (Figures 2A,C). The cell capability of differentiation and mineralization examined by Alizarin red staning (ARS) reduced by H_2_O_2_ was also attenuated by Cur (Figures 2B,D). Further, mRNA test showed the decreased expression of typical osteogenic marker genes (ALP, OCN, COL I, and Runx2) in OS injured model, were recovered after Cur administration (Figure 2E). Notably, no significant difference was obtained between the Cur group and NAC group.

### Cur Rescued the Expression of p-GSK3β and Nrf2 Decreased by H_2_O_2_

**FIGURE 3:**
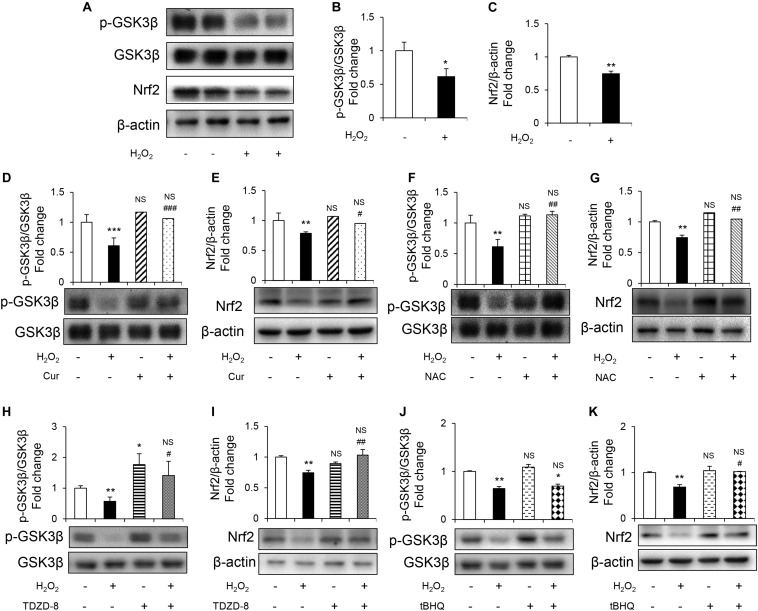
Cur attenuated H_2_O_2_-induced osteoblast apoptosis through GSK3β-Nrf2 signaling pathway. MC3T3-E1 cells were treated in the basic medium with or without H_2_O_2_ for 6 h. **(A)** Representative immunoreactive bands for phosphorylated GSK3β, GSK3β, and Nrf2 in MC3T3-E1 cells. **(B,C)** Quantification of immunoreactive bands for phosphorylated GSK3β relative to GSK3β and Nrf2 relative to β-actin. **(D,E)** Cur was added 24 h before H_2_O_2_. Densitometry of immunoreactive bands for p-GSK3β and GSK3β, Nrf2 and β-actin in MC3T3-E1 cells with (+) or without (–) Cur in the presence of H_2_O_2_ (+) or culture medium (–). **(F,G)** NAC was added 1 h before H_2_O_2_. Representative immunoreactive bands and relative levels of p-GSK3β and GSK3β, Nrf2 and β-actin in MC3T3-E1 cells with (+) or without (–) NAC treatment in the presence of H_2_O_2_ (+) or culture medium (–). Representative immunoblots are shown at the bottom. **(H,I)** TDZD-8 was added 1 h before H_2_O_2_. Representative immunoreactive bands and relative levels of p-GSK3β and GSK3β, Nrf2, and β-actin in MC3T3-E1 cells with (+) or without (–) TDZD-8 treatment in the presence of H_2_O_2_ (+) or culture medium (–). **(J,K)** tBHQ was added 1 h before H_2_O_2_. Representative immunoreactive bands and relative levels of p-GSK3β and GSK3β, Nrf2, and β-actin in MC3T3-E1 cells with (+) or without (–) tBHQ treatment in the presence of H_2_O_2_ (+) or culture medium (–). Data are presented as the mean ± SD from at least three independent experiments. *p < 0.05, **p < 0.01, ***p < 0.001, versus C group; NS, non-significantly different from C group. ^#^*p* < 0.05, ^##^*p* < 0.01, ^###^*p* < 0.001, versus the H_2_O_2_ group; ns, non-significantly different from H_2_O_2_ group.

To further study the mechanism underlying protective effect of Cur, GSK3β-Nrf2 signaling pathway was detected.GSK3β activity was evaluated by the level of phosphorylated GSK3β at inhibitory serine 9 residues (Romorini et al., 2016). It was shown that H_2_O_2_ reduced phosphorylation of GSK3β without changing the total GSK3β levels (Figures 3A,B). Nrf2 is the highly sensitive transcription factor of OS and its expression was downregulated induced by H_2_O_2_ (Figures 3A,C). Cur increased the levels of p-GSK3β (Figures 3D,F) and Nrf2 (Figures 3E,G) in H_2_O_2_ treatment group, so as the NAC treatment group. Appling TDZD-8, the specific GSK3β inhibitor, reversed the reduction of p-GSK3β and Nrf2 (Figures 3H,I), while using tBHQ, the activator of Nrf2, only increased the level of Nrf2 but not p-GSK3β (Figures 3J,K). These results indicated that GSK3β was the upstream regulator of Nrf2. Remarkably, the H_2_O_2_-induced inhibition of GSK3β-Nrf2 signaling pathway was reversed by Cur.

### GSK3β-Nrf2 Signaling Pathway Activating Protects MC3T3-E1 Cells From the Oxidative Damage Induced by H_2_O_2_

**FIGURE 4:**
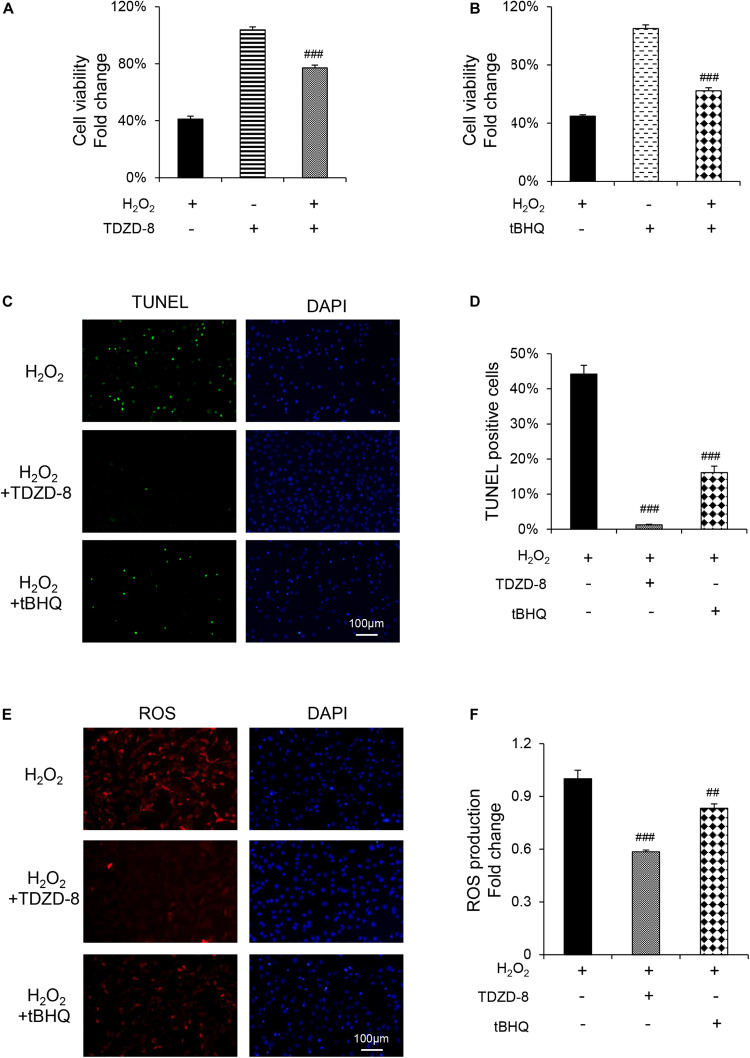
GSK3β-Nrf2 signaling pathway is involved in H_2_O_2_-induced apoptosis and ROS generation in MC3T3-E1 cells. MC3T3-E1 cells were treated with or without H_2_O_2_ in the basic medium for 6 h. TDZD-8 or tBHQ were added 1 h before H_2_O_2_. **(A)** Cell viability determined by MTT reduction in MC3T3-E1 cells with (+) or without (–) TDZD-8 treatment in the presence or absence of H_2_O_2_ (+). **(B)** Cell viability determined by MTT reduction in MC3T3-E1 cells with (+) or without (–) tBHQ treatment in the presence or absence of H_2_O_2_ (+). **(C,D)** The cells were immunostained for TUNEL (green). DAPI staining was used to mark the position of the nuclei. Scale bars = 100 μm. **(E,F)** The cells were harvested and stained with DCFH-DA (red). Scale bars = 100 μm. Data are presented as the mean ± SD from at least three independent experiments. *p < 0.05, **p < 0.01, ***p < 0.001, versus C group; NS, non-significantly different from C group. ^#^*p* < 0.05, ^##^*p* < 0.01, ^###^*p* < 0.001, versus the H_2_O_2_ group; ns, non-significantly different from H_2_O_2_ group.

**FIGURE 5:**
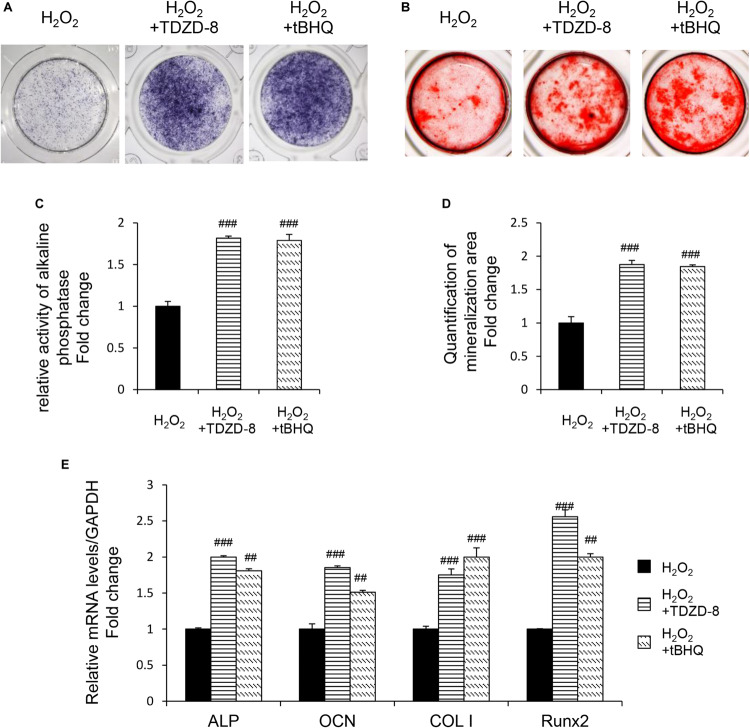
GSK3β-Nrf2 signaling pathway is involved in H_2_O_2_-induced osteoblast dysfunction. The MC3T3-E1 cells were treated in the osteogenesis differentiation medium with or without H_2_O_2_ for 6 h. TDZD-8 or tBHQ were added 1 h before H_2_O_2_. **(A)** After the cells cultured for 7 days and they were subjected to ALP staining in the indicated treatment groups. **(B)** The alizarin red staining of MC3T3-E1 cells showed the mineralizing matrix after cultured for 14 days. **(C)** ALP activity tested in MC3T3-E1 cells as indicated groups. **(D)** Mineralization capacity was evaluated by tests. **(E)** When cultured for 3 days the expression of osteogenic marker genes were analyzed. Data are presented as the mean ± SD from at least three independent experiments. *p < 0.05, **p < 0.01, ***p < 0.001, versus C group; NS, non-significantly different from C group. ^#^*p* < 0.05, ^##^*p* < 0.01, ^###^*p* < 0.001, versus the H_2_O_2_ group; ns, non-significantly different from H_2_O_2_ group.

TDZD-8 and tBHQ inhibited apoptosis induced by H_2_O_2_, as demonstrated by MTT test and TUNEL staining (Figures 4A–D). And the ROS homeostasis indicating H_2_O_2_-induced OS was reversed by TDZD-8 and tBHQ (Figures 4E–H). Furthermore, they also recovered the osteogenic differentiation ability, shown by ALP staining/activity and ARS (Figures 5A–D) as well as the expression of osteogenic genes (Figure 5E). All the results revealed that GSK3β-Nrf2 signaling pathway played a pivotal role in regulating osteoblasts apoptosis and dysfunction induced by OS.

## Discussion

Numerous studies have proved that OS-induced osteoblasts dysfunction plays an important role in the pathogenic progression of osteoporosis (Maggio et al., 2003; Baek et al., 2010). On the other hand, Cur, a classical antioxidant herb can efficaciously prevent and treat osteoporosis (Fu et al., 2015). However, the underlying molecular mechanism remains largely unveiled. In the present study, we found that OS significantly decreased osteoblasts viability and their osteoblastogenic differentiation. For the first time, we demonstrate that the suppression of GSK3β-Nrf2 signaling pathway could be a key molecular event accounting for these phenomena. We further showed that Cur effectively attenuated the oxidative damages and restored the differentiation of osteoblasts by retaining GSK3β-Nrf2 signaling.

Oxidative stress is characterized by the excessive generation of ROS, e.g., H_2_O_2_, O${}_{2}^{-}$, and ⋅OH (Hu et al., 2019). H_2_O_2_ is the most stable and common form of ROS, and is suitable to be used both as an intra- and an intercellular signal (Denisova et al., 2001). Thus, H_2_O_2_ is considered to be a widely accepted initiator of cell oxidative injury model (Xu et al., 2011; Liang et al., 2013; Fu et al., 2015). In this study, we found that H_2_O_2_ could result in significantly increased ROS production, apoptosis as well as decreased cell viability in MC3T3-E1 cells. These results were in line with our previous findings that H_2_O_2_ exerted a profound cytotoxic effect on a human osteoblast-like cell line (Dai et al., 2017). We further showed that H_2_O_2_ also downregulated the osteoblastogenic differentiation, which was reflected by the significant reduction of ALP activity, calcium deposition and osteogenic genes’ expression. These findings were consistent with the previous reports that the suppressed bone regeneration in humans and animals was mainly caused by reduced osteoblast number and hindered osteogenic function (Marie and Kassem, 2011).

Curcumin can trap radicals to exert its antioxidant function, thereby being used to prevent and treat chronic metabolic diseases (Goel et al., 2008), such as osteoporosis (Hussan et al., 2012; Cho et al., 2013). Results of this study showed that the administration of Cur significantly attenuated cell toxicity effects of H_2_O_2_. DCFH-DA staining showed that, similar as NAC – a classical exogenous antioxidant, Cur could dramatically alleviate cytosolic ROS level induced by H_2_O_2_. In our previous study, we also demonstrate that Cur effectively decreases mitochondrial ROS and restores consequence dysfunction in human osteoblast-like cells (Chao et al., 2017). These findings suggested that Cur was a powerful and cell-organelle-non-specific antioxidant to suppress endogenous ROS production. Furthermore, in a non-OS microenvironment, Cur could not significantly influence cell viability and osteogenic functions. Consequently, the protective effect of Cur on osteoblasts was most likely attributed to its antioxidant property but not to its stimulatory effect on osteoblastogenesis. This character of Cur may partly explain why Cur is mostly used in primary or secondary osteoporosis (Hussan et al., 2012; Chen et al., 2016) other than bone formation under normal conditions. On the other hand, previous studies also show that Cur inhibits the proliferation and differentiation of human or rat osteoblasts (Notoya et al., 2006; Moran et al., 2012). These inconsistencies may be due to the varieties of cell types and different concentrations of Cur used in different studies. For example, Cur might lead to the apoptosis of MG-63 osteosarcoma cells at low concentrations, while it did not affect the viability of human osteoblasts at the same condition (Chang et al., 2014, 2015). Therefore, a further exploration needs to be performed to figure out the exact mechanisms for these phenomena.

Glycogen synthase kinase 3β is a rate-limiting enzyme of glycogen synthesis and acts as an important negative regulator of bone metabolism (Qi et al., 2015). Heterozygous GSK3β deficient mice exhibit increased bone formation (Noh et al., 2009). Pharmacological antagonists of GSK3β prevent skeletal unloading or ovariectomy-induced bone loss in mice (Warden et al., 2010; Zahoor et al., 2014). In the present study, H_2_O_2_ could dramatically decrease GSK3β phosphorylation (Ser 9) and lead to its activation. The inactivation of GSK3β by TDZD-8 not only attenuated H_2_O_2_-induced apoptosis, but also restored the osteogenic activity of the osteoblasts. Our previous study indicated that GSK3β inhibitor mitigated mitochondrial dysfunction of diabetic mice hippocampal (Huang et al., 2015) and Saos-2 cells (Dai et al., 2017), thereby alleviating oxidative damages. This finding suggested that GSK3β played a key role in the OS-induced osteoblasts injury. Cur significantly restored the level of p-GSK3β that was reduced by H_2_O_2_. Furthermore, Cur treatment also decreased ROS and protected osteoblasts from OS-damages. These results suggested that Cur might protect osteoblasts against H_2_O_2_-induced disturbance though the redox homeostasis-regulating function of GSK3β pathway.

Nuclear factor erythroid 2 related factor 2 has also been reported to play an important role in the regulation of bone metabolism. Nrf2-deficient mice exhibit a loss in BMD in femora and a reduction in cortical bone area in vertebrae (Ibanez et al., 2014). Our previous study demonstrates that Nrf2 downregulation is closely related to the enhanced alveolar bone loss in diabetic periodontitis in rats (Li et al., 2018). Moreover, the deletion of Nrf2 suppresses antioxidant enzymes and elevates the intracellular ROS level in osteoblasts, which significantly compromises their ability to differentiate and mineralize (Rana et al., 2012). In the present study, our results also found that the expression of Nrf2 significantly decreased in OS injury model of osteoblasts. The activation of Nrf2 by tBHQ, a specific chemical agonist of Nrf2, restored both cell viability and osteogenic activity. Notably, Cur efficaciously restored the expression of Nrf2, which alleviated OS and consequently attenuated H_2_O_2_-induced damages of osteoblasts. As a major regulatory transcription factor, Nrf2 responds to various cellular stresses and regulates the transcription of broad range of antioxidant genes. More recently, it is reported that Nrf2 and its downstream genes opposes transcriptional up-regulation of apoptosis-related genes (Lin et al., 2014; Pellegrini et al., 2016). The decreased apoptosis rate in our results suggested that the protective effect of Cur could be, at least partially, attributed to Nrf2 activation. On the other hand, our results showed that Cur increased the level of osteoblastogenic genes including Runx2, a transcription factor regulates other osteoblast-related genes (e.g., ALP, OCN, COL I) (Bialek et al., 2004; Komori, 2010), in osteoblasts under the exposure to H_2_O_2_. It is well recognized that Runx2 plays a key role in osteoblastic differentiation and bone formation. Nrf2 is reported to positively regulate the expression of Runx2 (Sim et al., 2016). Therefore, our finding suggested that Nrf2 stabilization and activation mediated the protective effects of Cur on the viability and differentiation of osteoblasts.

**FIGURE 6:**
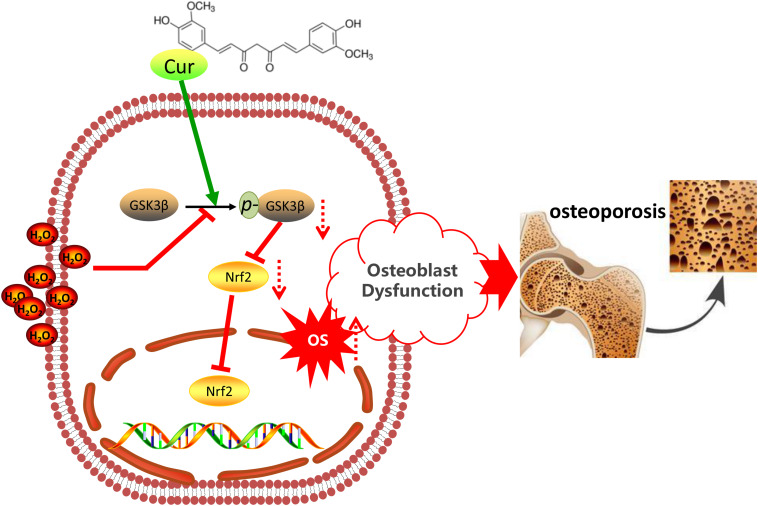
Schematic diagram of the mechanism of Cur in OS-induced osteoblasts’ dysfunction.

Nuclear factor erythroid 2 related factor 2, as a cell signaling sensor, can be negatively regulated by GSK3β (Liu et al., 2017; Cuadrado et al., 2018). In this study, TDZD-8 treatment significantly increased the expression of Nrf2, while tBHQ did not restore the expression of p-GSK3β. This finding suggested that Nrf2 was the downstream molecule of GSK3β. GSK3β-Nrf2 signaling pathway plays a crucial role in regulating the redox homeostasis and the downstream cell behavior (Lv et al., 2017; Yi et al., 2018). In this study, we, for the first time, demonstrated that Cur pretreatment significantly antagonized the destructive effects of H_2_O_2_ so as to enhance the level of p-GSK3β as well as Nrf2 expression. The enhancement of GSK3β-Nrf2 signaling was correlated to the reduced ROS level, decreased apoptosis, and retained cell differentiation and mineralization. Consistent with the results in this study, inactivation of GSK3β by TDZD-8 or activation of Nrf2 by tBHQ also protect osteoblasts from H_2_O_2_-mediated oxidative damages. Our finding suggested that Cur protected osteoblasts from H_2_O_2_-induced OS damages were most likely mediated by its capacity of retaining GSK3β-Nrf2 signaling pathway (Figure 6).

There are still some limitations in this study. We only adopted a mouse preosteoblast cell line. More primary cultured osteoblasts or cell lines are needed to affirm the mechanism underlying the protective effect of Cur on OS induced osteoblasts dysfunction. On the other hand, further *in vivo* studies should be arranged to verify the preventive effect of Cur on osteoporosis and corroborate the role of GSK3β-Nrf2 singling pathway.

## Conclusion

Glycogen synthase kinase 3β-nuclear factor erythroid 2 related factor 2 activation and ROS scavenging could be the key mechanism responsible for Cur’s pro-survival and differentiation-promoting actions in H_2_O_2_ induced oxidative damage of MC3T3-E1 cells. Cur may act as a potential bone-protective therapeutic agent for the prevention or treatment of osteoporosis and effectively stimulate bone regeneration under OS pathological condition.

## Data Availability Statement

The datasets generated for this study are available on request to the corresponding author.

## Author Contributions

XL, SH, GW, and JM conceived and designed the study. XL, YC, YM, PD, XZ, HC, YW, and IB carried out the experiments and collated the data. XL and YC wrote the original draft. XS, SH, GW, and TF reviewed and edited the manuscript. All authors have read and approved the final submitted manuscript.

## Conflict of Interest

The authors declare that the research was conducted in the absence of any commercial or financial relationships that could be construed as a potential conflict of interest.
